# Progressive alveolar echinococcosis after discontinuation of anthelmintic therapy

**DOI:** 10.1186/1756-3305-6-287

**Published:** 2013-10-01

**Authors:** Michael Meilinger, Christina Stoeckl, Marion Pollheimer, Peter Kern, Emil C Reisinger, Katharina Seeber, Robert Krause, Holger Flick, Martin Hoenigl

**Affiliations:** 1 Division of Pulmonology, Department of Medicine, Medical University of Graz, Auenbruggerplatz 20, 8036 Graz, Austria; 2 Section of Infectious Diseases and Tropical Medicine, Medical University of Graz, Auenbruggerplatz 15, 8036 Graz, Austria; 3 Department of Pathology, Medical University of Graz, Auenbruggerplatz 25, 8036 Graz, Austria; 4 WHO Informal Working Group on Echinococcosis, Comprehensive Infectious Diseases Center, Universitätsklinikum Ulm, Albert-Einstein-Allee 23, 89081 Ulm, Germany; 5 Abteilung für Tropenmedizin und Infektionskrankheiten, Universitätsmedizin Rostock, Ernst-Heydemann-Str. 6, 18057 Rostock, Germany

**Keywords:** Alveolar echinococcosis, *Echinococcus multilocularis*, Albendazole, Anthelmintic therapy, Liposomal amphotericin B

## Abstract

We report a case of a female patient with alveolar echinococcosis (AE) who presented with progressive pulmonary and hepatic lesions and had a fatal outcome. AE affecting the liver, the lungs and the brain had been diagnosed 20 years ago and treated successfully with albendazole and stereotactic gamma knife therapy. Due to severe hair loss albendazole was stopped 14 years before presentation. Lesions had remained stable in imaging studies for at least 11 years, but then had started to progress. Lifelong anthelmintic maintenance therapy and regular follow-up may therefore be crucial in order to prevent such a dramatic clinical course.

## The Case

A 49-year-old Swiss female was admitted in December 2012 with progressive dyspnoea and bilateral peripheral oedema to the Division of Pulmonology, University Hospital of Graz, Austria. Following the diagnosis of alveolar echinococcosis (AE) affecting the liver, the lungs and the brain, in 1992 treatment with mebendazole plus interferon gamma was initiated [1]. Then, in 1994, therapy was switched to albendazole and stereotactic gamma knife therapy due to progressive cerebral lesions. As the patient remained stable with minimal neurological symptoms we concluded that gamma knife radiosurgery may be an alternative for patients with cerebral AE for whom surgery is not possible [2].

After four years of stable disease anthelmintic treatment was stopped in 1998 due to severe hair loss, which was mentally unbearable for the patient. The patient did not receive anthelmintic treatment for the following 14 years. Until September 2009 the patient was followed-up monthly and then annually with pulmonary, hepatic and cerebral lesions remaining stable in follow–up imaging studies. The patient had no further complaints. After 2009 the patient was, however, lost to follow-up, and no further imaging studies were performed at our centre.

**Figure 1:**
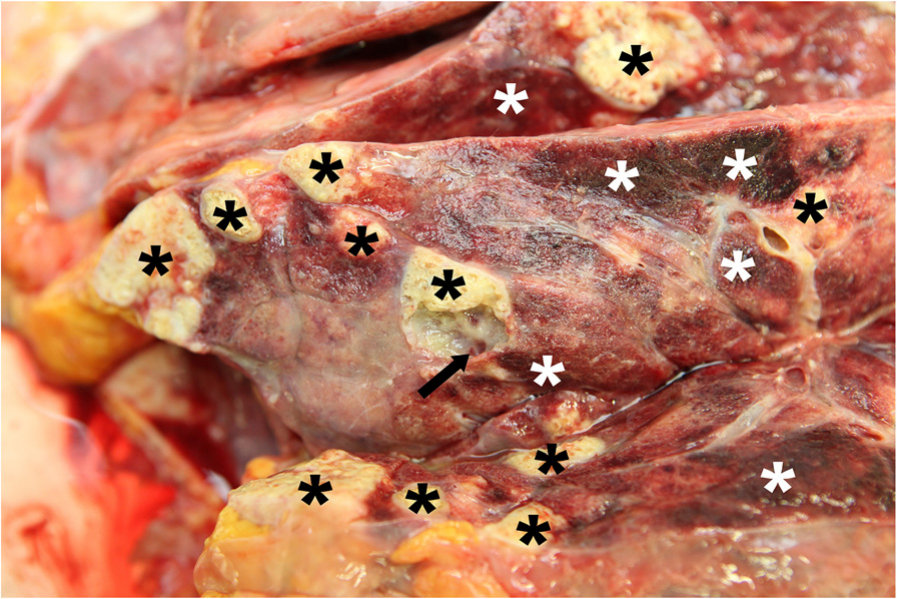
**Macroscopic appearance of the affected lung.** Cystic lesions are indicated by the arrow, the black asterices indicate calcified and the white asterices indicate hemorrhagic necrotic lesions.

At admission in 2012 computed tomography revealed a dramatic progress of the known pulmonary bilobular lesions when compared to 2009 and a large partially calcified hepatic lesion with complete obstruction of the inferior vena cava, while cerebral lesions had remained stable in magnetic resonance imaging of the brain. According to the recommendations of the WHO-Informal Working Group on Echinococcosis, albendazole (400 mg b.i.d.) was initiated [3-5]. After four days the patient developed severe respiratory failure and was intubated. Liposomal amphotericin B (3 mg/kg daily) was added as anthelmintic salvage therapy. Despite using all technical facilities the patient died of respiratory insufficiency after 20 days at the intensive care unit. Autopsy confirmed cystic, partly calcified and centrally necrotized infiltrations of both lungs (Figure 1), the liver and the brain. Histology of the brain, the lung and the liver revealed large cystic lesions with eosinophilic brood capsules, scolices and daughter cysts. The cysts were surrounded by dense fibrovascular tissue with chronic inflammatory cells and showed a varying degree of calcification.

## Discussion

Caused by *Echinococcus multilocularis,* AE is endemic in Central and Eastern Europe, the Near East, Russia, China, and Japan [6,7]. Increasing annual incidence rates are reported from a number of countries, including Austria [8]. After an asymptomatic incubation period, patients typically develop a slowly progressive tumor-like destructive liver disease. Since the introduction of mebendazole in 1977 and albendazole in 1992 life expectancy has dramatically improved [9]. Ammann and colleagues previously reported a 67-year-old male patient with non-resectable AE of the liver who had been continuously treated for 13 years with mebendazole until he died due to a condition unrelated to AE. Autopsy findings were suggestive of a parasitocidal efficacy of mebendazole treatment [10]. The fact that pulmonary and hepatic lesions remained stable for at least 11 years in our patient, even though anthelmintic therapy was discontinued, suggests that intensified treatment has a huge impact on *Echinococcus multilocularis*. The following progression of AE with the fatal outcome, however, stands in contrast to a potential curative efficacy. To prevent such dramatic clinical courses, lifelong anthelmintic maintenance therapy and lifelong regular follow-up seems to be crucial [11].

## Abbreviations

AE: Alveolar echinococcosis.

## Competing interests

The authors declare that they have no competing interests.

## Authors’ contributions

HF, MM, CS, RK, ER, PK, KS and MH treated the patient and provided the clinical data; MP and MM provided autopsy photographs; MM, KS, CS, ER, RK MP and PK edited the report. HF and MH drafted the first version of the report. All authors read and approved the final manuscript.

## Authors’ information

Peter Kern is Chairman of the WHO Informal Working Group on Echinococcosis. Emil Reisinger is now Head of the Department of Tropical Medicine, Infectious Diseases and Nephrology, University of Rostock, Rostock, Germany. Ten years before he had been at the Medical University of Graz and was responsible for treating the patient between 1992 and 2003. All other authors are physicians at the Medical University of Graz (Section of Infectious Diseases and Tropical Medicine, Division of Pulmonology, Department of Pathology) who were involved in the case in 2012. 
